# A dataset for bilateral lower limb muscle synergy patterns in unilateral anterior cruciate ligament reconstructed patients

**DOI:** 10.1038/s41597-026-07543-2

**Published:** 2026-06-01

**Authors:** Hongxiang Zhang, Mengli Wei, Yaping Zhong, Gewenjin Zhu

**Affiliations:** 1 Wuhan Sports University, Wuhan, 430070 China; 2 Hubei Sports and Health Innovation and Development Research Center, Wuhan, 430070 China; 3 Soochow University, Suzhou, 215021 China

## Abstract

This data descriptor presents a bilateral lower limb muscle synergy dataset from 17 unilateral anterior cruciate ligament reconstruction athletes (9 men, 8 women; training experience > 4 years; national level one or two athletes) at 6 months post-surgery during stable gait testing. A cross-sectional study design was adopted. Surface electromyography signals from eight key muscles of both lower limbs were processed using non-negative matrix factorization to extract muscle synergy structures. The dataset comprehensively documents: (1) basic participant information; (2) K-STARTS functional test results; (3) gait mechanics parameters (gait speed, center of mass mediolateral displacement velocity, and center of pressure mediolateral displacement velocity); and (4) core muscle synergy indicators, including synergy weight matrix (W), activation coefficient matrix (H), number of synergies, muscle contribution weights, time to peak activation, activation level of synergy, proportion of significant activation duration, overall synergy activation level, and co-activation duration. This dataset provides systematic, reproducible, and standardized data support for analyzing bilateral lower limb neuromuscular control differences following anterior cruciate ligament reconstruction, and can be directly applied to symmetry analysis, functional assessment, and individualized rehabilitation research.

## Introduction

Anterior cruciate ligament (ACL) rupture is a frequent and severe athletic injury that immediately compromises knee stability and can abruptly threaten an athlete’s career^1,2^. Arthroscopic anterior cruciate ligament reconstruction (ACLR) is the current gold-standard treatment, intended to restore knee function and accelerate return to sport. Nevertheless, the risk of second injury remains substantial: among athletes who do return, the re-rupture rate on the operated side reaches 7%, while the cumulative incidence for both ipsilateral and contralateral knees in athletes under 25 years old climbs to 23%^1^.

Gait is the most fundamental and frequently performed human movement pattern and is tightly governed by central neural control; any lower-limb injury or sensorimotor disturbance can therefore precipitate measurable gait abnormalities^3^. After ACLR, patients commonly exhibit asymmetric gait secondary to reduced muscle strength, restricted joint range of motion, and impaired proprioception. Typical deviations include quadriceps-avoidance patterns and altered loading-response times^3–5^. Symmetry reflects the balanced capacity of both limbs in terms of strength, coordination, and neuromuscular control; its loss produces compensatory strategies and abnormal load distribution that elevate the risk of re-injury^6^. Consequently, gait symmetry is widely regarded as a key indicator of locomotor function in both pathological and healthy populations^7^.

Muscle synergy refers to the coordinated activation of muscle groups that allows the central nervous system to control movement through a small set of control variables rather than by regulating individual muscles^8,9^. The regularity of these synergy modules determines movement coordination and stability^10^; therefore, a systematic evaluation of synergy-based activation patterns during gait in ACLR patients is essential for understanding the mechanisms underlying their motor deficits.

By supplying open-access muscle synergy data, this dataset fills a critical gap in shared resources, enabling a detailed characterization of bilateral lower limb synergy patterns in individuals after unilateral ACLR. It offers an evidence base for gait-function assessment and individualized rehabilitation training, thereby helping to reduce the risk of re-injury.

## Materials and Methods

### Experimental Participants

The data in this study were used for paired analysis. A priori sample size estimation was performed using G*Power 3.1 software, with a medium effect size of d = 0.8^11^, significance level α = 0.05, and statistical power 1-β = 0.80. The calculated minimum required sample size was 15 participants.

This study ultimately recruited 17 students from Wuhan Sports University (9 males, 8 females, all at China national first or second level). Inclusion criteria: aged 18–25 years; unilateral ACLR performed ≥ 6 months earlier; autograft: bone–patellar-tendon–bone or hamstring tendon; long-term sport participation (training experience > 4 years)with national grade-2 (or higher) certification; no post-operative infection or other surgical complications. Exclusion criteria were: concomitant lower-limb fracture, posterior cruciate ligament rupture, or other major tissue injuries; Magnetic Resonance Imaging evidence of an ACL avulsion fracture at the insertion site; history of two or more ACLR procedures or lower-limb fracture within the past year. All testing was conducted at the Sports Big Data Research Center of Wuhan Sports University. All subjects signed informed consent forms, agreeing to participate in the experiment, data collection, and the use of their anonymized data for academic research and public release. The 17 recruited subjects were either first or second level athletes, all recruited subjects underwent arthroscopic autologous bone-patellar tendon-bone surgery. The basic information of the study subjects is shown in Table 1.

**Table 1:** Basic Information of Subjects.

Parameter	Overall (n = 17)	Male (n = 9)	Female (n = 8)
Age (years)	20.94 ± 1.30	20.89 ± 1.62	21.00 ± 0.93
Height (cm)	172.24 ± 6.27	177.51 ± 2.86	166.31 ± 2.16
Weight (kg)	64.81 ± 11.44	74.42 ± 6.20	54.00 ± 1.73
Years of training (years)	5.77 ± 1.35	6.33 ± 1.32	5.13 ± 1.13
Months post-surgery (Months)	7.94 ± 1.64	7.11 ± 1.05	8.88 ± 1.73
K-START score	10.67 ± 2.92	11.15 ± 3.41	10.13 ± 2.36

### Experimental Protocol

**Fig. 1:**
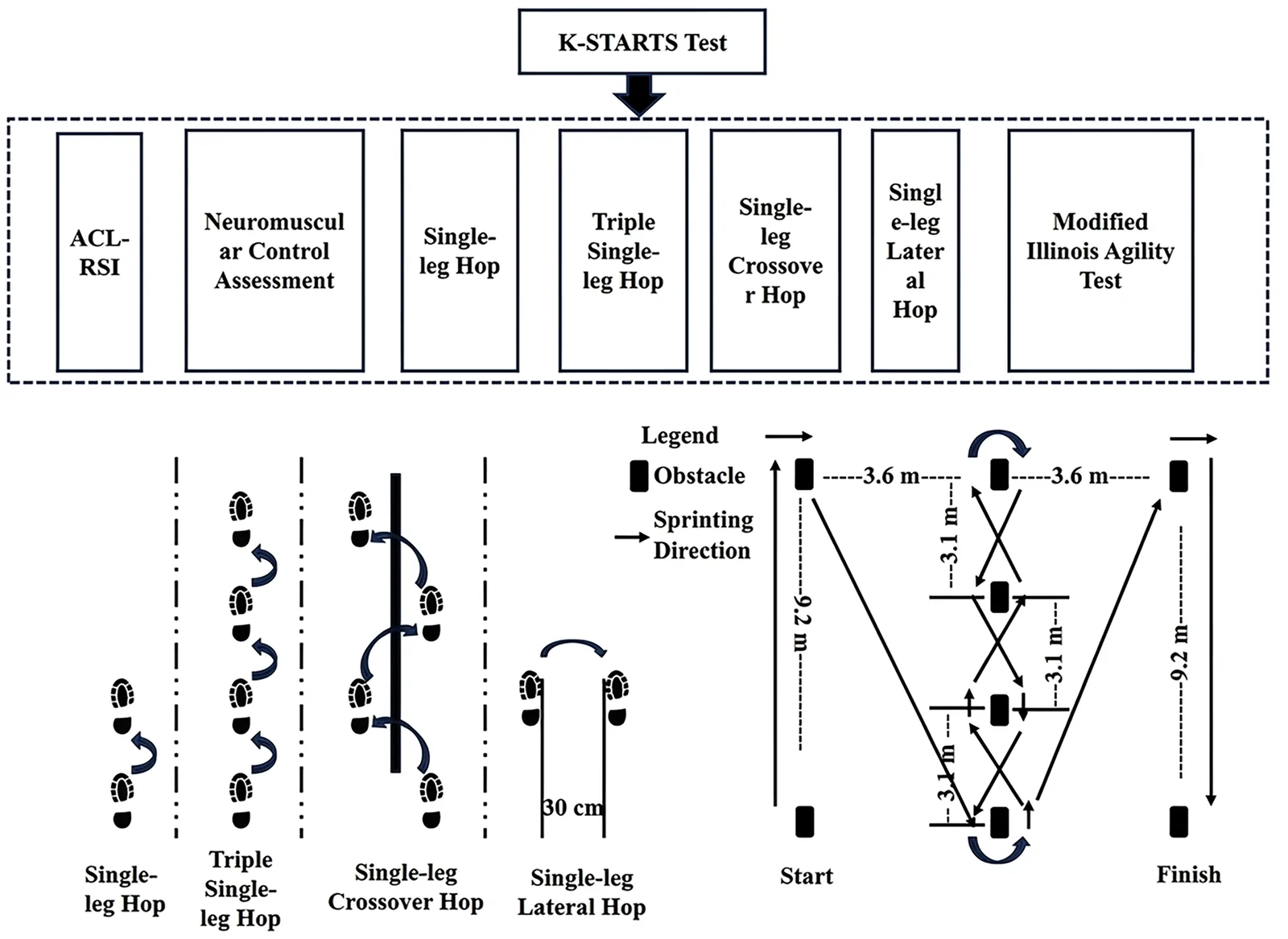
K-STARTS Test.

The K-STARTS test was first conducted to assess the psychological and functional recovery status following ACL injury. All procedures of the K-STARTS test were supervised by two sports rehabilitation specialists, who were responsible for recording subjects’ test responses and evaluating each test item. The test comprised two components: psychological assessment and functional testing. Subjects first completed the psychological scale (ACL-RSI), followed by functional testing in a designated area. Functional test items included neuromuscular control assessment, single-leg hop, triple single-leg hop, single-leg crossover hop, single-leg lateral hop, and modified illinois agility test. During the testing period, two high-definition cameras continuously recorded subjects’ movement performance. Each functional test task was performed consecutively for 3 trials, with 2 minutes of rest between each test item. If subjects experienced discomfort or pain during testing, the test item was immediately terminated. See Fig. 1.

**Fig. 2:**
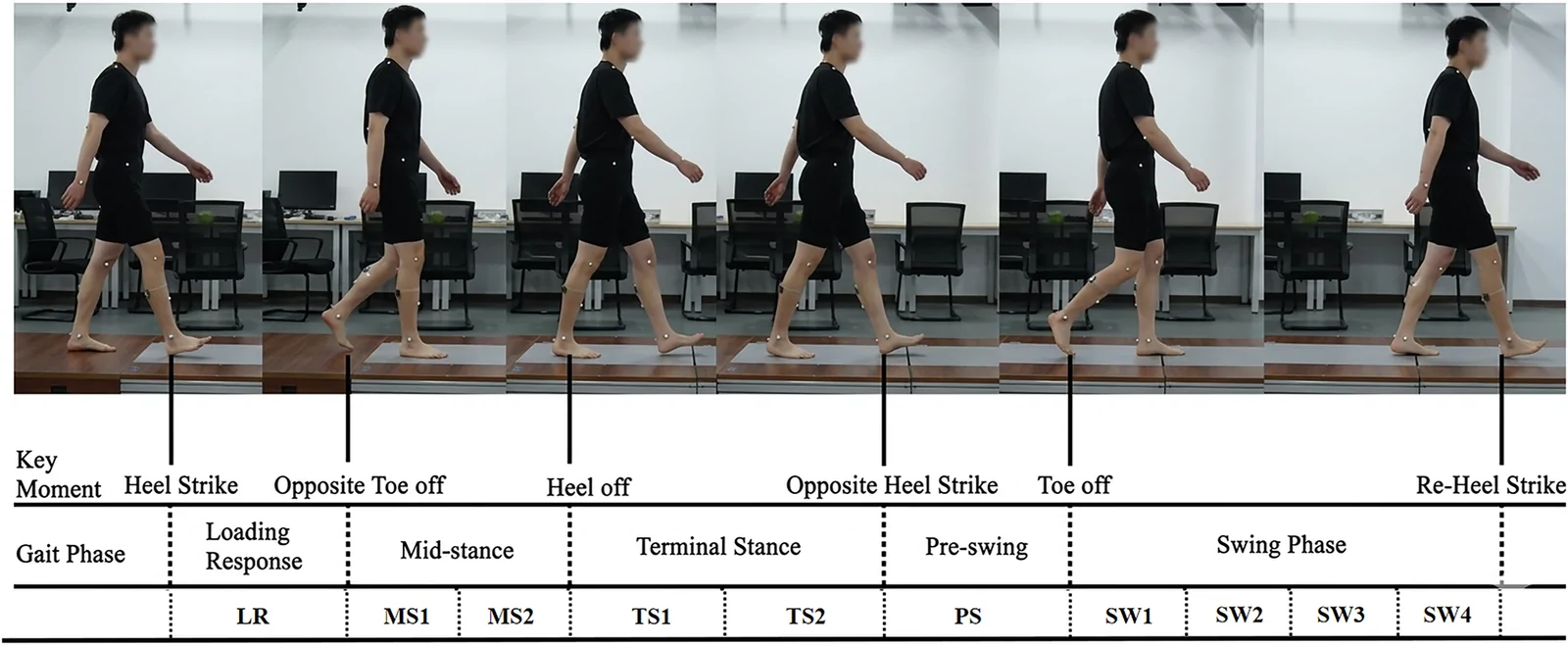
Division of Phases in Stable Gait Testing.

Participants wore a standard black fitted top and walked barefoot. A stable gait protocol was used: subjects looked straight ahead, walked at their customary speed, and were instructed not to deliberately alter step length or speed. Six full gait cycles were recorded for each limb. For analysis, the gait cycle was divided into five functional phases and ten equal sub-regions^12^ (each 10% of the cycle): Loading response (LR) – 0–10%Mid-stance (MS) – 10–30%, split into MS1 (10–20%) and MS2 (20–30%); Terminal stance (TS) – 30–50%, split into TS1 (30–40%) and TS2 (40–50%);Pre-swing (PS) – 50–60%; Swing phase (SW) – 60–100%, split into SW1 (60–70%), SW2 (70–80%), SW3 (80–90%), and SW4 (90–100%). See Fig. 2.

### Data Collection

A three-dimensional motion capture system (SIMI, Germany) was used. After testing, the video data (100 Hz) were imported into the motion capture system analysis software. A three-dimensional force plate (Kistler, Switzerland) was used to collect kinetic data, with a sampling frequency of 1000 Hz. The Delsys Trigno™ wireless surface electromyography system (Delsys, USA) was used. Wireless sEMG sensors were precisely applied to the subjects, following the electrode placement guidelines of Seniam^13^. This study recorded EMG signals from the vastus lateralis (VL), rectus femoris (RF), vastus medialis (VM), biceps femoris (BF), semitendinosus (ST), tibialis anterior (TA), gastrocnemius medialis (GM), and gastrocnemius lateralis (GL). The wireless sEMG sampling frequency was set at 2000 Hz.

### Data processing

The three-dimensional force plate data were analyzed to identify the initial contact and toe-off events during gait. Initial contact was defined as the moment when the vertical ground reaction force exceeded 20 N, while toe-off was defined as the moment when the vertical ground reaction force fell below 20 N^14^. The sampling frequency was 1000 Hz. Kinetic data were filtered using a Butterworth low-pass filter with a cutoff frequency of 50 Hz^13^.

Kinematic data were obtained with a three-dimensional motion-capture system (SIMI, Germany). Following data collection, the video recordings were imported into the system’s analysis software for processing. Kinematic data were filtered using a Butterworth low-pass filter with a cutoff frequency of 10 Hz^15^.

Based on the aforementioned force plate and motion-capture system, gait characteristic parameters were extracted across three dimensions: gait speed, center of mass mediolateral displacement velocity, and center of pressure mediolateral displacement velocity. Specifically, gait speed was calculated as the average forward velocity based on the horizontal displacement of the center of mass and gait cycle duration. The center of mass displacement velocity was determined by calculating the rate of change in three-dimensional center of mass position using body segment parameters, reflecting dynamic balance control capability. The center of pressure displacement velocity was computed from the vertical reaction forces and moments to assess the movement velocity of the center of pressure during the stance phase, serving as an indicator of plantar pressure modulation efficiency.

The EMG signals were first processed with a band-pass filter of 10–500 Hz and then full-wave rectified. The rectified signals were enveloped using a 4th-order low-pass Butterworth filter with a cut off frequency of 10 Hz^16^.

Referring to previous research^17^, the signals were normalized using the maximum value of each signal. The normalized EMG signal amplitude represents the proportional peak amplitude, with values ranging from 0-1 (dimensionless). The time axis was normalized to the 0% to 100% interval through linear interpolation to ensure temporal comparability across signals of different durations^18^.

During the muscle synergy analysis process, researchers collected surface electromyography (EMG) signals from eight target muscles (VM, RF, VL, BF, ST, GL, GM, TA) and processed them into an 8 × 101-dimensional feature matrix E (where m = 8 represents the number of muscles, and n = 101 is the length of the time series). The muscles are listed in the order mentioned above. Subsequently, the non-negative matrix factorization algorithm^19^ (Formula 1) was employed to decompose this matrix into: a k-dimensional synergy matrix W, which characterizes the muscle synergy patterns, and an activation coefficient matrix H, which reflects the temporal activation features. The feature matrix E′ was reconstructed via matrix multiplication (W × H) (Formula 2) for subsequent analysis.

1 $$E={\sum }_{i=1}^{N}{W}_{i}{H}_{i}+e\,{W}_{i}\ge 0,{H}_{i}\ge 0$$

2 $${E}^{{\prime} }={\sum }_{i=1}^{N}{W}_{i}{H}_{i}\,{W}_{i}\ge 0,{H}_{i}\ge 0$$

Note: *N* is the number of muscle synergies, and *e* is the residual.

Subsequently, key synergistic features were extracted from the original feature matrix E and its reconstructed muscle activation pattern matrix E′. The specific analysis metrics and evaluation criteria are as follows:

1. Number of muscle synergies involved: The variance accounted for (VAF) analysis method was employed to compare the variance and its rate of change (Formula 3) between the reconstructed feature matrix E′ and the original matrix E. The criterion for determining the number of synergies involved was a VAF value greater than 95%^20^.
  3 $${VAF}=1-\frac{{\sum }_{i=1}^{m}{\sum }_{j=1}^{n}{\left({E}_{{ij}}-{E}_{{ij}}^{{\prime} }\right)}^{2}}{{\sum }_{i=1}^{m}{\sum }_{j=1}^{n}{\left({E}_{{ij}}\right)}^{2}}$$
2. Muscle Relative Weight. After decomposing the original matrix E, the contribution degree of each muscle can be obtained, allowing for comparison of the differences in contributions among muscles within each synergy^21^.
3. Time to Peak Activation of Muscle Synergies. The coefficient matrix H, obtained from decomposing the original matrix E, reflects the activation intensity of each synergy. The time at which the activation intensity of each muscle synergy reaches its peak is recorded^22^.
4. Activation Level of Muscle Synergies and Overall Activation Level. The coefficient matrix H can also be used to evaluate the activation intensity of each synergy as well as the overall activation level of the entire synergy structure^23^.
5. Duration of Significant Activation and Co-activation of Muscle Synergies. The duration of significant activation for a muscle synergy refers to the period during which its activation level exceeds 50% of its maximum activation. Co-activation refers to the proportion of time during which two or more muscle synergies are simultaneously active.

## Data Records

The muscle synergy and electromyography dataset generated from this study has been deposited in the FigShare public data repository^24^. All published data have been de-identified and are publicly available under the Creative Commons Attribution 4.0 International License (http://creativecommons.org/licenses/by/4.0/). The dataset can be accessed at: https://doi.org/10.6084/m9.figshare.30928694.

This dataset provides comprehensive muscle synergy and electromyographic data from 17 anterior cruciate ligament reconstructed patients during stable gait cycles, comprising four synergy module files—“Affected Side Synergy Module 1”, “Affected Side Synergy Module 2”, “Healthy Side Synergy Module 1”, and “Healthy Side Synergy Module 2”—where Page 1 of each file contains the muscle weight matrix W showing relative contributions of vastus lateralis, rectus femoris, vastus medialis, biceps femoris, semitendinosus, tibialis anterior, medial gastrocnemius, and lateral gastrocnemius, Page 2 gives the associated muscle activation matrix H, and Page 3 reports time to peak activation, Activation Level of Synergy, and Proportion of Significant Activation Duration, together with two overall synergy parameter files for affected and healthy limbs respectively—each with Page 1 listing overall synergy parameters and Page 2 showing VAF value changes across varying synergy numbers—as well as two raw EMG data files capturing affected side and healthy side muscle electrical activity from the same eight muscles across 17 subjects (ID1–17), and a “Basic Information and Kinematic Data” file providing age, ACL Return to Sport after Injury index score, gait speed, and other clinical covariates, enabling integrated analyses of modular neuromuscular control organization, inter-limb asymmetries, and their relationships with functional recovery outcomes in post-ACLR rehabilitation.

## Technical Validation

The quality of kinematic data was ensured through a multi-level validation process. During data collection, the motion-capture cameras were calibrated and validated prior to data collection to ensure that errors remained within acceptable limits. In the event of marker detachment during a trial, the detached markers were replaced, followed by a new static calibration. Marker trajectory reconstruction was performed using the proprietary software of the motion-capture system (SIMI, Germany). All three-dimensional marker trajectories were subjected to frame-by-frame visual inspection to exclude instances of marker misidentification or trajectory jumps.

During electromyography data collection, surface electromyography is subject to considerable variability if sensor equipment, placement locations, and positioning procedures are not standardized. Variability was minimized by adopting the recommended procedures of the SENIAM (Surface ElectroMyoGraphy for the Non-Invasive Assessment of Muscles) guidelines. A wireless surface electromyography system (Delsys Trigno™, USA) was used. Electrodes were placed on dry, shaved skin, oriented along the direction of muscle fibers. This placement was performed by the same experienced researcher for all participants. Electrodes were positioned at the longitudinal midpoint between the motor end plate and the tendon, as described by SENIAM. Participants were asked to contract their muscles to verify correct positioning. The placement of wireless sensors avoided cable stretching, and sensors were securely fixed with double-sided adhesive tape and covered with additional tape. All procedures conformed to SENIAM guidelines to ensure reliable and accurate electromyography data collection.

### Mechanical Data of the healthy and affected Limbs

This section provides supplementary reports on the comparative results of gait mechanical parameters between the unaffected and affected limbs. Through this information, the biomechanical characteristic consistency of the dataset and potential inter-limb differences can be assessed across three dimensions: gait speed, center of mass displacement velocity, and center of pressure displacement velocity. See Table 2.

**Table 2:** Mechanical Data of the healthy and affected Limbs.

Parameter	Healthy	Affected
Gait speed (m/s)	0.376 ± 0.046	0.370 ± 0.046
Center of mass mediolateral displacement velocity (m/s)	0.029 ± 0.011	0.029 ± 0.009
Center of pressure mediolateral displacement velocity (m/s)	0.052 ± 0.012	0.054 ± 0.014

### Reference synergy data for the healthy and affected limbs

**Fig. 3:**
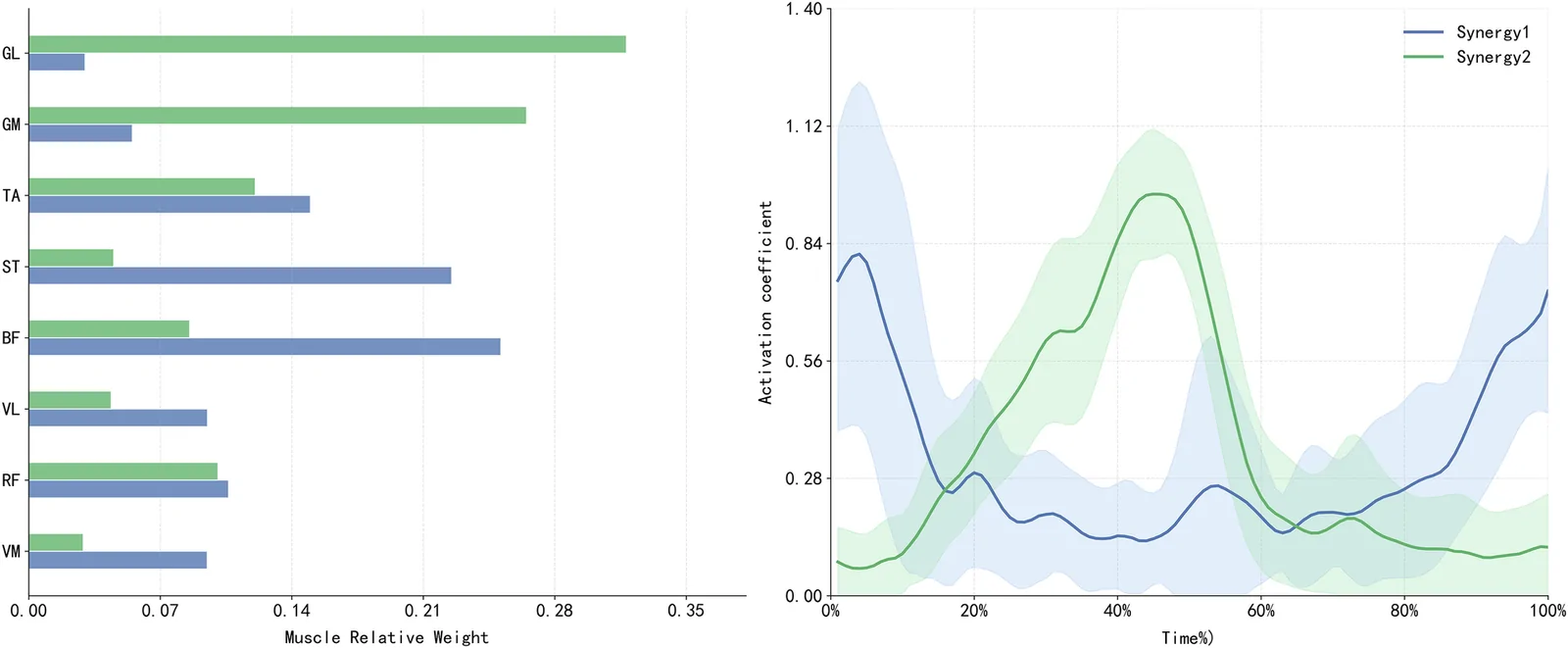
Synergy Matrix W and Activation Coefficient Matrix H for the healthy side.

**Fig. 4:**
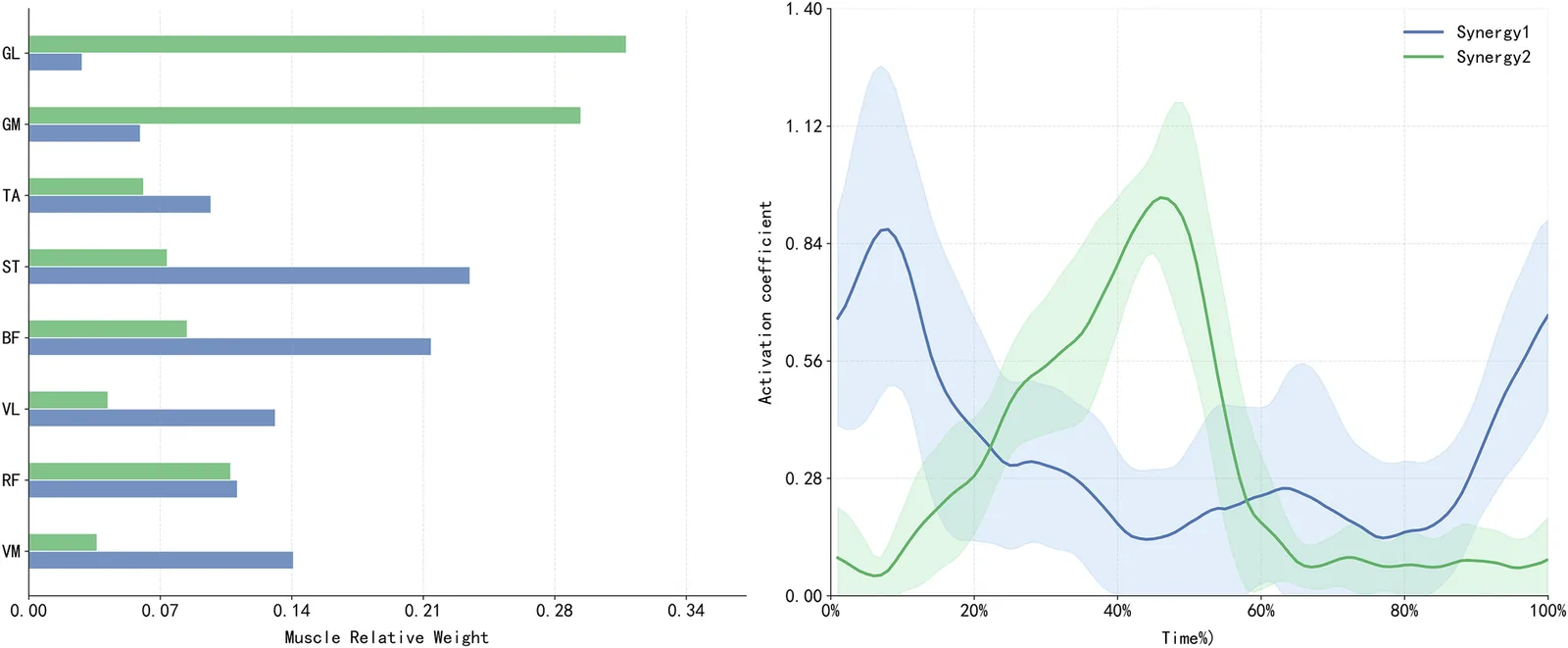
Synergy Matrix W and Activation Coefficient Matrix H for the affected side.

In this dataset, the reference muscle synergies for the stable gait test consisted of 2 synergies for both the unaffected and affected limbs. However, overall, two sets of muscle synergies could be extracted under the unaffected and affected conditions (see Fig. 3 and Fig. 4). Specifically, Synergy 1 was dominated by the gastrocnemius lateralis and gastrocnemius medialis; Synergy 2 was dominated by the semitendinosus and biceps femoris. Figure Legend: Left panel–Synergy matrix W: Each bar represents the relative weighting of each muscle within a given synergy. The horizontal axis indicates the percentage contribution (0–100%) of each muscle to the synergy. The vertical axis lists the eight muscles examined: Vastus Lateralis (VL), Rectus Femoris (RF), Vastus Medialis (VM), Biceps Femoris (BF), Semitendinosus (ST), Tibialis Anterior (TA), Gastrocnemius Medialis (GM), and Gastrocnemius Lateralis (GL). Right panel–Activation coefficient matrix H: The horizontal axis represents normalised gait cycle time (0–100%, from heel strike to next heel strike). The vertical axis represents the activation coefficient (dimensionless, normalised amplitude). Solid lines indicate the mean across participants, and shaded areas represent the range (minimum to maximum) of activation patterns.

### Variation in synergy number between healthy and affected limbs

**Fig. 5:**
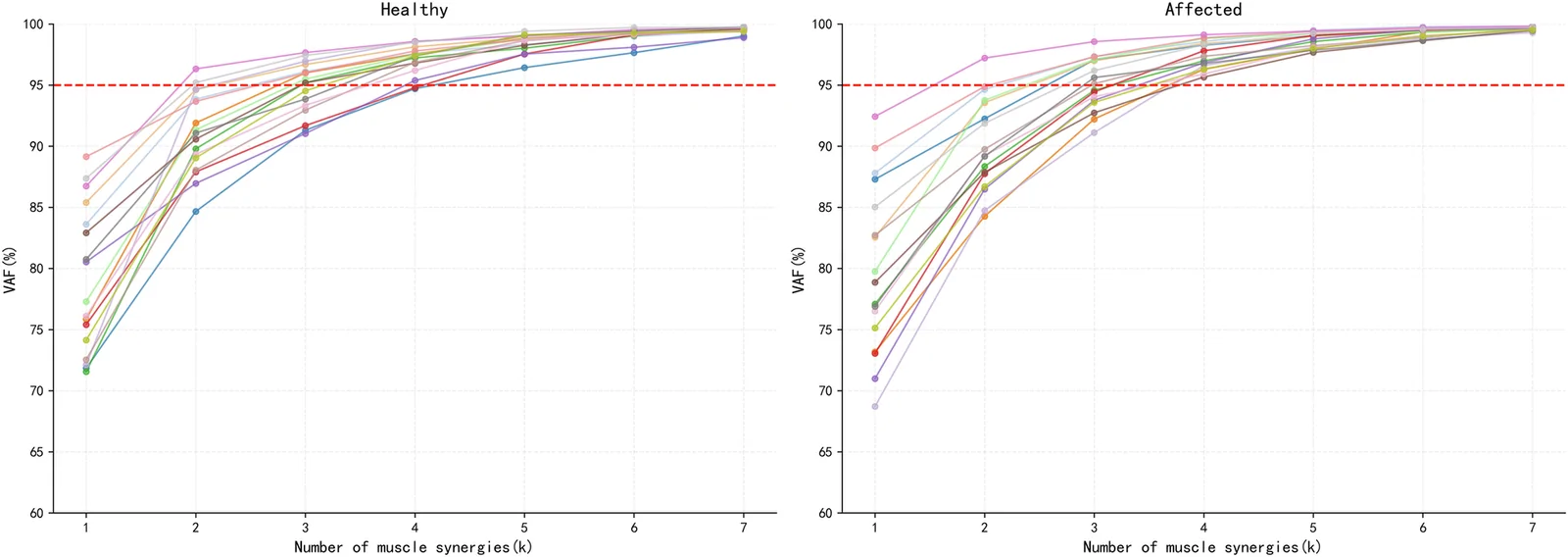
Variation of VAF values on the healthy and affected sides with the number of synergies.

When the VAF threshold was set above 95%, the number of muscle synergies at the individual level was mostly 3 for both the unaffected and affected limbs, whereas when the number of synergies increased to 5, the VAF exceeded 95% for all individuals (see Fig. 5). It should be noted that the aforementioned results represent the number of synergies at the individual level, reflecting the degree of neuromuscular control refinement and individual differences among participants. To enable meaningful comparison of synergy patterns across different participants, this study extracted 2 classes of reference synergies through cluster analysis (see Figs. 3 and 4).

### Muscle Relative Weight of the Synergies

In the stable gait assessment, some variations were observed in the lower limb muscle synergy weights between the unaffected and affected sides of anterior cruciate ligament reconstruction patients. In Synergy 2, the weight of the tibialis anterior on the affected side decreased substantially compared to the unaffected side, from 0.120 ± 0.106 to 0.060 ± 0.050, while the weights of the remaining muscles also exhibited varying degrees of change (see Table 3 for details). It should be noted that the aforementioned description is based solely on numerical observations from the current dataset, without inferential statistical comparisons. Data users may conduct subsequent statistical analyses according to their research purposes.

**Table 3:** Muscle Relative Weight of the Synergies for the Affected and Unaffected Limbs.

Synergy Type	Muscle	Healthy	Affected
Synergy 1	VL	0.094 ± 0.088	0.139 ± 0.072
	RF	0.106 ± 0.075	0.109 ± 0.050
	VM	0.094 ± 0.080	0.129 ± 0.078
	BF	0.249 ± 0.117	0.211 ± 0.116
	ST	0.223 ± 0.102	0.231 ± 0.109
	TA	0.149 ± 0.112	0.095 ± 0.059
	GM	0.055 ± 0.067	0.058 ± 0.075
	GL	0.030 ± 0.026	0.028 ± 0.030
Synergy 2	VL	0.029 ± 0.026	0.036 ± 0.033
	RF	0.100 ± 0.056	0.106 ± 0.054
	VM	0.044 ± 0.043	0.041 ± 0.041
	BF	0.085 ± 0.081	0.083 ± 0.075
	ST	0.045 ± 0.044	0.073 ± 0.063
	TA	0.120 ± 0.106	0.060 ± 0.050
	GM	0.263 ± 0.046	0.289 ± 0.060
	GL	0.316 ± 0.063	0.313 ± 0.071

Note: “N/A” indicates missing data.

### Synergy Activation Coefficient

In the stable gait assessment, some variations were observed in the lower limb synergy activation coefficients between the unaffected and affected sides of anterior cruciate ligament reconstruction patients. For example, in Synergy 2, the activation level of synergy of the affected side was 0.303 ± 0.048, while that of the unaffected side was 0.344 ± 0.079, with the affected side numerically lower than the unaffected side. The remaining indicators were relatively similar between the two sides (see Table 4 for details). It should be noted that the aforementioned description is based solely on numerical observations from the current dataset, without inferential statistical comparisons. Data users may conduct subsequent statistical analyses according to their research purposes.

**Table 4:** Differences in Muscle Synergy Parameters Between the Affected and Healthy Limbs.

Indicator	Synergy	Healthy	Affected
TTP (%)	Synergy 1	35.625 ± 40.664	24.250 ± 29.540
	Synergy 2	45.688 ± 3.516	45.625 ± 5.005
ALS	Synergy 1	0.316 ± 0.099	0.343 ± 0.101
	Synergy 2	0.344 ± 0.079	0.303 ± 0.048
PSAD (%)	Synergy 1	22.875 ± 10.269	23.250 ± 12.102
	Synergy 2	28.250 ± 11.767	24.062 ± 7.514
OSAL	N/A	0.321 ± 0.063	0.320 ± 0.054
CoActD (%)	N/A	8.294 ± 7.824	10.176 ± 8.553

Note: TTP (Time to peak Activation); ALS (Activation Level of Synergy); PSAD (Proportion of Significant Activation Duration); OSAL (Overall Synergy Activation Level); CoActD (Co-activation Duration); “N/A” indicates missing data.

### Limitations

The present data come from a single group exploratory design. One concern is that the observed side to side differences might simply reflect limb dominance rather than the effects of ACLR. However, Arauz *et al*.^25^ reported that healthy adults walking at a self-selected speed show no significant inter-limb differences or asymmetrical patterns between the dominant and non-dominant legs, making this explanation unlikely. Nevertheless, future work should incorporate larger samples and more rigorous randomized controlled trials to confirm these findings.

### Ethics Statement

The study protocol was approved by the Medical Ethics Committee of Wuhan Sports University (approval No. 2023029) and registered with the Chinese Clinical Trial Registry (registration No. ChiCTR2400083640).

### Consent

Each subject gave written informed consent.

## Data Availability

The data can be accessed at: https://doi.org/10.6084/m9.figshare.30928694.

## Code availability

This study does not contain any original code.
